# Labeling Compliance of Dietary Supplements: An Observational Study in the Beni Mellal Khenifra Region

**DOI:** 10.1002/fsn3.71512

**Published:** 2026-02-04

**Authors:** Aziz Galman, Mourad Chikhaoui, Fahd A. Nasr, Mohamed Bouhrim, Mohammed Al‐zharani, Ashraf Ahmed Qurtam, Hassan A. Rudayni, Hassan Alahyane, Rachid Lotfi, Hind Belamgharia, Morad Kaddouri, Charaf Dlimi, Naoual Moulahid, Mohamed Reda Kachmar, Khalid Boutoial

**Affiliations:** ^1^ Laboratory of the Engineering and Applied Technologies, Higher School of Technology Sultan Moulay Slimane University Beni Mellal Morocco; ^2^ High Institute of Nursing Professions and Health Techniques Beni Mellal Morocco; ^3^ Laboratory of Ecology and Environment, Faculty of Sciences Ben M'sik Hassan II University Casablanca Morocco; ^4^ Biology Department, College of Science Imam Mohammad Ibn Saud Islamic University (IMSIU) Riyadh Saudi Arabia; ^5^ Biological Engineering Laboratory, Faculty of Sciences and Techniques Sultan Moulay Slimane University Beni Mellal Morocco; ^6^ Laboratories TBC, Laboratory of Pharmacology, Pharmacokinetics, and Clinical Pharmacy Faculty of Pharmaceutical and Biological Sciences Lille France; ^7^ Integrative Biochemistry and Neuroscience for Health and the Environment Laboratory, Polydisciplinary Faculty, Department of Biology Sultan Moulay Slimane University Beni Mellal Morocco; ^8^ Human Nutrition, Bioactives and Oncogenetics Team, Faculty of Science Moulay Ismail University Meknes Morocco

**Keywords:** compliance, dietary supplements, labeling, nutrivigilance

## Abstract

The increasing consumption of dietary supplements among physically active individuals raises concerns about labeling compliance and consumer safety, particularly in Morocco, where data remain limited. This study assessed the labeling compliance of dietary supplements with national and international regulations in the Beni Mellal–Khénifra region. An observational study was conducted between December 2024 and May 2025 on 403 dietary supplements collected from fitness centers, parapharmacies, and supermarkets. Products were evaluated using a 30‐item regulatory checklist, and data were analyzed using Chi‐squared tests and Spearman correlations. Overall, 81.39% of supplements were non‐compliant. The highest non‐compliance was observed in creatine and amino acid‐based products (100%) and multivitamins (86.2%), while medicinal plant‐based supplements showed lower non‐compliance (48%). Missing regulatory information, including dietary category, energy value, safety warnings, and registration numbers, was significantly associated with non‐compliance (*p* < 0.001). Imported products were significantly less compliant than locally produced ones (*r* = −0.694, *p* < 0.001). The high prevalence of labeling non‐compliance highlights the urgent need for strengthened regulatory enforcement and market surveillance to improve consumer protection and labeling transparency.

## Graphical abstract

An evaluation of 403 dietary supplements marketed in the Beni Mellal–Khénifra region revealed widespread labeling non‐compliance (81.39%), with the highest rates observed in creatine‐ and amino acid–based products. Imported supplements were significantly less compliant than locally produced ones, emphasizing the need for stronger regulatory enforcement and improved market surveillance to ensure consumer safety.

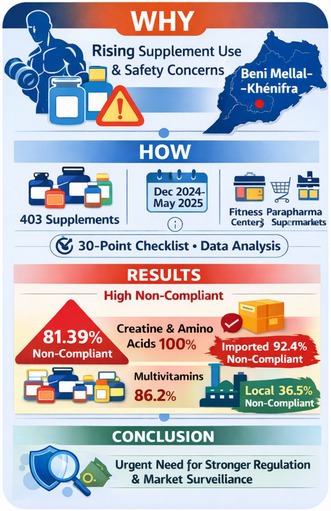

## 1 Introduction

Dietary Supplements (DS) are defined as concentrated sources of nutrients or other substances with nutritional or physiological effects intended to supplement the normal diet (EPCEU 2002). These include vitamins, minerals, herbs, proteins, amino acids, enzymes, and other active compounds. Their popularity has significantly increased in recent decades, particularly in industrialized societies, due to their reputation for safety and presumed health benefits (Grand View Research 2023). Notable examples include the role of vitamin D in supporting recovery from influenza and COVID‐19, folic acid in reducing the risk of stroke among hypertensive individuals, and omega‐3 fatty acids in lowering cardiovascular mortality in older adults (Hasan et al. 2024; Walrand 2018). Athletes represent one of the largest consumer groups of dietary supplements globally (Devaux and Brisard 2016; Abreu et al. 2023; Wang et al. 2023). These products are commonly used to enhance athletic performance (Maughan et al. 2018), accelerate post‐exercise recovery, promote muscle development (Baharirad et al. 2025), and support weight management (Mah et al. 2022). Global dietary supplement consumption is rising worldwide, raising public health concerns and highlighting the need for regulatory frameworks (Bardou‐Boisnier and Caillaud 2015). In Morocco, this trend reflects broader nutritional transitions driven by urbanization and globalization (Allali 2017). Studies report that 36%–57% of Moroccans use supplements, with higher prevalence among women and educated young adults (El Kartouti and Khalfaoui 2020; Touijri et al. 2024). Main motivations include correcting deficiencies (42.6%) and enhancing functions (36.4%), with multivitamins being most consumed (80%). Healthcare professionals are the primary advisors (46.7%), and pharmacies the main distribution channel (78.7%). Despite growth, overall use remains modest relative to potential benefits and daily nutritional needs (Touijri et al. 2024).

However, the excessive use of dietary supplements has been frequently associated with health risks (Hassan et al. 2020). These risks are exacerbated when products are contaminated with prohibited substances such as trace metals, anabolic androgenic steroids, clenbuterol, and sibutramine (French Agency for Food, Environmental and Occupational Health, and Safety 2016). Several studies have also demonstrated that certain supplements may contain illicit doping agents or undeclared contaminants, potentially leading to serious adverse health effects (Marcotrigiano et al. 2018; Kozhuharov et al. 2022; Galman et al. 2024). Notably, the ANSES has reported cases of severe adverse reactions and fatalities linked to the use of sports‐related supplements (French Agency for Food, Environmental and Occupational Health, and Safety 2016). In the United States, numerous studies on dietary supplement consumption have reported a variety of adverse effects (Tarn et al. 2013). Similarly, research conducted in Japan has identified a comparable impact, albeit with a lower reported incidence among consumers (Chiba et al. 2015). These discrepancies may be attributed to the underreporting of adverse events by users in both countries, limiting accurate assessment of the true prevalence.

In Morocco, only one study to date has focused on the monitoring of adverse effects related to dietary supplement consumption (Galman et al. 2024). This study reported that users experienced several adverse effects, including myalgia, gastralgia, palpitations, and insomnia. These findings underscore the urgent need to implement a structured monitoring system and to conduct in‐depth research on the safety of dietary supplements. To ensure the safety of dietary supplements, Moroccan authorities have established a health surveillance system for food products, including dietary supplements. This responsibility is shared between the Ministry of Agriculture, Sea Fishing, Rural Development, Water and Forests (MASFRDWF) (Ministry of Agriculture, Sea Fishing, Rural Development, Water and Forests 2009), and the Ministry of Health and Social Protection (MHSP 1974). In this regulatory framework, the marketing of dietary supplements, whether through official distribution channels (e.g., pharmacies) or public outlets (e.g., supermarkets, parapharmacies, gyms), raises concerns regarding compliance with national and international standards, particularly in terms of product quality and counterfeit risks. These challenges highlight the urgent need to reinforce regulatory oversight and health controls by the relevant authorities (Ministry of Health and Social Protection, and Ministry of Agriculture, Sea Fishing, Rural Development, Water and Forests 2023). In this context, the question that arises is whether there is, in fact, a rigorous and operational system in place for the monitoring and regulation of the marketing and use of dietary supplements. This issue encompasses several key aspects: on the one hand, the compliance of labeling with current regulatory standards, ensuring reliable and transparent information for consumers; on the other hand, the detection and prevention of counterfeiting, which poses a major threat to public health; and finally, the existence and effectiveness of adverse effect reporting systems, which are essential for implementing a genuine nutrivigilance framework aimed at identifying, documenting, and preventing health risks associated with the consumption of these products. In this regard, the present study aimed to assess the labeling compliance (LC) of the most commonly consumed products among the athletic population who participated in our study carried out in 2024 at Beni Mellal city (Galman et al. 2024), by comparing them to the standards defined by the Food and Agriculture Organization (FAO) and World Health Organization (WHO) (Food and Agriculture Organization and World Health Organization 2024). This investigation is conducted within fitness centers where these dietary supplements are marketed and used by fitness practitioners.

## 2 Material and Methods

### 2.1 Study Design and Area

The present study aims to evaluate the LC of dietary supplements distributed within the Beni Mellal‐Khénifra region. This observational, descriptive, and analytical study was carried out between December 2024 and May 2025 across 60 fitness centers located in five cities: Beni Mellal, Khenifra, Khouribga, Azilal, and Fquih Ben Salah.

### 2.2 Sampling Strategy and Sample Size

In light of our previous research highlighting the widespread use of proteins, amino acids, vitamins, and medicinal plants extracts among fitness club users (Galman et al. 2024), a purposive sampling strategy was implemented in the current study. A total of 403 dietary supplement products were collected from diverse retail outlets, including fitness centers, parapharmacies, and supermarkets. These products were subsequently organized into three principal categories (Table 1).

**TABLE 1:** Different categories of dietary supplements sampled.

Categories	Total
Protein and amino acid supplements	279
Vitamin‐based supplements	99
Medicinal Plants‐based supplements	25

### 2.3 Survey and Data Collection

A structured observation checklist comprising 30 criteria (C1–C30) was developed in accordance with Moroccan regulatory standards and international guidelines to assess the labeling compliance (LC) of dietary supplements (EPCEU 2002; Ministry of Health and Social Protection, and Ministry of Agriculture, Sea Fishing, Rural Development, Water and Forests 2023; Food and Agriculture Organization and World Health Organization 2024; EPCEU 2006; EPCEU 2011). The checklist covered mandatory labeling requirements, health warnings, legibility, traceability, and the disclosure of active ingredients. The compliance assessment was adapted according to product category: supplements containing vitamins and proteins/amino acids were evaluated using 26 criteria, excluding C18, C24, C25, and C26, whereas medicinal plant–based supplements were assessed using 28 criteria, excluding C18 and C24, which are specific to cannabis‐related products.

To ensure data validity and reliability, observations were conducted in pairs by five trained auditors who had received dedicated training on national and international food labeling regulations. Data collection relied on direct observation across several distribution channels, including parapharmacies, fitness centers, and supermarkets, while pharmacies were excluded. For each product, a standardized observation form was completed in accordance with Moroccan regulations, notably Law 09–08 on the protection of personal data to ensure anonymity and Law 31–08 guaranteeing consumer rights. A predefined coding system was applied (Table 2). Each sample was assigned a unique identification code (ID) and classified into one of three categories: vitamins, proteins, or plant extracts. The manufacturing origin of each product was recorded as either imported or locally produced. The date of observation and other relevant information were systematically documented. Observer codes were assigned according to the city in which the samples were collected. All procedures complied with ethical research principles, including obtaining informed consent from retail outlet managers, and strict confidentiality of commercial data was maintained.

**TABLE 2:** Coding of site cities for monitoring compliance with labeling regulations.

Observer code	Collection city
BM‐compliance	Beni Mellal
AZ‐compliance	AZilal
KH‐compliance	KHenifra
FBS‐compliance	Fquih Ben Salah
KG‐compliance	KhouribGa

### 2.4 Statistical Analysis

All data were entered and analyzed using IBM SPSS Statistics version 25. The distribution of variables was tested using Kolmogorov–Smirnov and Shapiro–Wilk tests, revealing significant departures from normality (*p* < 0.001). Consequently, non‐parametric methods were applied. The analysis included Chi‐squared tests for bivariate associations and Spearman's rank correlations.

## 3 Results

### 3.1 Dietary Supplement and Compliance Labeling

Off the 403 dietary supplement samples analyzed, 328 products (81.39%) exhibited non‐compliant labeling (Table 3). The highest non‐compliance rate was observed among protein and amino acid‐based supplements, reaching 92.47%. Notably, specific ingredients such as creatine, casein, arginine, taurine, methionine, and tryptophan demonstrated a 100% non‐compliance rate. Supplements containing whey protein, glutamine, branched‐chain amino acids (BCAAs), and collagen also showed high levels of non‐compliance, with rates ranging from 81.25% to 97.82%. Vitamin‐based supplements presented a non‐compliance rate of 58.59%, which increased to 86.20% in the case of multivitamin formulations. In contrast, medicinal plant extract‐based products showed a comparatively lower non‐compliance rate of 48%, indicating better labeling adherence relative to other categories of dietary supplements (Table 3).

**TABLE 3:** Categories of dietary supplements and their non‐compliance labeling.

Category	Total (*n*)	Absolute frequency of non‐compliance products (*n*)	Relative frequency of non‐compliance products (%)
Vitamins	99	58	58.59
Monovitamin	20	15	75
Bivitamin	30	10	33.33
Trivitamin	20	8	40
Multivitamin	29	25	86.20
Proteins/Amino acids	279	258	92.47
Whey	46	45	97.82
Casein	12	12	100
Creatine	94	94	100
Glutamine	35	33	94.28
BCAA (Branched‐Chain Amino acids)	18	17	94.44
Arginine	15	15	100
Taurine	7	7	100
Methionine	9	9	100
Tryptophan	13	13	100
Collagen	16	13	81.25
Keratin	14	10	71.42
Medicinal plants	25	12	48
Total (*n*)	403	328	81.39

### 3.2 Labeling Compliance Evaluation Criteria

Table 4 presents the percentage of criteria applied to evaluate the LC of dietary supplements. Certain criteria demonstrated full compliance, notably the readability of the product name (C1), the use of a non‐misleading brand name (C2), the indication of net quantity (C3), the listing of ingredients in descending order (C6), and the use of appropriate measurement units (C7), each achieving a 100% compliance rate. For several other criteria, high rates of labeling non‐compliance were observed, particularly for the presence of a dietary substitution disclaimer (C11), declaration of botanical identity (C25), specification of the plant part used (C26), and compliance with bilingual labeling requirements (C30), with non‐compliance rates of 78.91%, 94.04%, 94.04%, and 72.95%, respectively. Moderate levels of non‐compliance were also recorded for the presence of warnings for at‐risk groups (C14), indication of the CE registration number (C16), display of the ONSSA authorization number (C17), and listing of adverse effects (C29), with non‐compliance rates of 55.58%, 52.36%, 55.09%, and 60.3%, respectively.

**TABLE 4:** Frequency of observed compliance criteria of sampling product.

Labeling criteria (C)	Compliant		Non‐compliant	
	Absolute frequency (*n*)	Relative frequency (%)	Absolute frequency (*n*)	Relative frequency (%)
Trade name is legible, understandable, and indelible (C1)	403	100	0	0
The commercial name is not misleading by suggesting a therapeutic use or resembling the name of drug (C2)	403	100	0	0
Net quantity is clearly indicated (C3)	403	100	0	0
Category “Dietary supplement based on…” is mentioned (C4)	279	69.23	124	30.77
Quantity of nutrients is visibly stated (C5)	402	99.75	1	0.25
List of ingredients ordered by descending mass (C6)	403	100	0	0
Correct units of measurement are used (C7)	403	100	0	0
Statement “Dietary supplement, not a medication” is indicated (C8)	195	48.39	208	**51.61**
Recommended Daily Allowance (RDA) is indicated (C9)	345	85.61	58	14.39
Energy value is specified (C10)	306	75.93	97	24.07
Statement “Does not replace a balanced diet” is included (C11)	85	21.09	318	**78.91**
Clear instructions for use are provided (C12)	401	99.5	2	0.5
Precautions for use are indicated (C13)	370	91.81	33	8.19
Warning “Keep out of reach of children” is present (C14)	179	44.42	224	**55.58**
Warnings or indications for at‐risk groups (e.g., pregnant women, children) are mentioned (C15)	256	63.52	147	36.48
Registration number (DMP‐MHPS) is stated (C16)	192	47.64	211	**52.36**
ONSSA approval number (for Moroccan products) is stated (C17)	181	44.91	222	**55.09**
ANRAC authorization number is indicated (relevant for cannabis extracts, e.g., cannabidiol) (C18)	403	100	0	0
Name and business name of the manufacturer are mentioned (C19)	403	100	0	0
Name and business name of the marketing authorization holder are mentioned (C20)	193	47.89	210	**52.11**
Batch number, manufacturing/expiration dates, and storage conditions are included (C21)	399	99.01	4	0.99
Measured concentrations are < 10% of the declared amounts (C22)	394	97.77	9	2.23
Measured concentrations are > 20% of the declared amounts (C23)	394	97.77	9	2.23
Tetrahydrocannabinol (THC) concentration is mentioned (C24)	403	100	0	0
Botanical identity of the plant is provided (C25)	24	5.96	379	**94.04**
Specific plant part used is mentioned (C26)	24	5.96	379	**94.04**
Health claims are stated (C27)	391	97.02	12	2.98
Nutritional claims are stated (C28)	279	69.23	124	30.77
Adverse effects are described (C29)	160	39.70	243	**60.30**
Information is provided in both Arabic and French (C30)	109	27.05	294	**72.95**

*Note:* The bold values are used to underscore the dominant or most significant findings across the dataset.

### 3.3 Correlation Between Supplement Combination, Market Source, and Labeling Conformity

Pearson's correlation analysis revealed a weak but statistically significant negative correlation between supplement formulation type (single‐ingredient vs. combined) and commercial origin (*r* = −0.115, *p* = 0.021), suggesting that combined formulations are more frequently produced locally, whereas imported products are predominantly single‐ingredient. Additionally, a strong and highly significant negative correlation was observed between commercial origin and LC (*r* = −0.694, *p* < 0.001), indicating that imported supplements exhibit lower compliance with labeling regulations compared to locally produced ones. In contrast, no statistically significant association was found between formulation type and LC (*r* = 0.078, *p* = 0.116), although a slight positive trend suggests that combined formulations may be marginally more compliant (Table 5).

**TABLE 5:** Correlation matrix between combination, commercial origin, and labeling compliance of dietary supplements.

	Combination	Commercial origin	Labeling compliance
Combination	—	−0.115 (*p* = 0.021)	0.078 (*p* = 0.116)
Commercial origin	−0.115 (*p* = 0.021)	—	−0.694 (*p* < 0.001)
Labelling compliance	0.078 (*p* = 0.116)	−0.694 (*p* < 0.001)	—

### 3.4 Compliance Study of Vitamin Supplement Labeling

The analysis of LC across different types of vitamin supplements reveals notable variations (Table 6). Bivitamin and trivitamin products demonstrated significantly lower non‐compliance rates, with 33.3% and 40% respectively, compared to other categories. Statistical analysis confirmed the significance of these differences, with *χ* ^2^ values of 61.197 and 29.941 (*p* < 0.001 for both), supported by Fisher's exact test. This suggests that bivitamin and trivitamin supplements tend to adhere more closely to labeling regulations. In contrast, monovitamin and multivitamin supplements showed higher rates of labeling non‐compliance, at 75% and 86.20%, respectively. However, these differences were not statistically significant, as indicated by *p*‐values of 0.343 and 1.000, respectively.

**TABLE 6:** Association between type of dietary supplement and labeling compliance among sampled products.

Type of dietary supplement	Non compliance		*χ* ^2^	*p*
	*n*	%		
Monovitamin	15	75	1.224	0.343
Bivitamin	10	33.30	61.197	0.000
Trivitamin	8	40	29.941	0.000
Multivitamin	25	86.20	0.126	1.000

### 3.5 Compliance Study of the Labeling of Protein Supplements, Amino Acids, and Medicinal Plants

The results of the chi‐squared (*χ* ^2^) and Fisher's exact tests assessing LC of protein‐based, amino acid‐based, and medicinal plants derived dietary supplements are summarized in Table 7. Statistically significant non‐compliance was observed for creatine‐based supplements (100% non‐compliant; *χ* ^2^ = 23.576; *p* < 0.001), whey protein products (97.82% non‐compliant; *χ* ^2^ = 7.476; *p* = 0.004), and medicinal plant‐based supplements (48.0% non‐compliant; *χ* ^2^ = 25.352; *p* < 0.001). In contrast, although certain supplements such as glutamine, arginine, and tryptophan exhibited high non‐compliance rates (≥ 94%), these differences were not statistically significant, likely due to limited sample sizes or reduced variability (Table 7). These findings suggest that products containing isolated proteins and amino acids, particularly creatine and whey, are far more prone to labeling irregularities compared to medicinal plants dietary supplements, warranting increased regulatory scrutiny for this product category.

**TABLE 7:** Compliance of samples of protein supplements, amino acids, and medicinal plants.

DS	Non‐compliance		*χ* ^2^	*p*
	*n*	%		
Whey	45	97.82	7.476	0.004
Casein	12	100	2.379	0.228
Creatine	94	100	23.576	0.000
Glutamine	33	94.28	3.073	0.093
BCAA	17	94.44	1.557	0.329
Arginine	15	100	2.996	0.144
Taurine	7	100	1.370	0.604
Methionine	9	100	1.770	0.366
Tryptophan	13	100	2.583	0.140
Collagen	13	81.25	0.085	0.730
Keratin	10	71.42	1.660	0.256
Medicinal plants excluding cannabis	12	48	25.352	0.000

### 3.6 Correlational Study on the Compliance of Creatine‐Based Supplements

The correlation analysis between individual labeling criteria and the overall compliance of dietary supplements identified several significant associations, as summarized in Table 8. Criterion C4 demonstrated a significant negative correlation with LC (*χ* ^2^ = 47.753, *p* < 0.001, *r* = −0.344), indicating that the presence of this label is frequently associated with non‐compliant products. Similarly, C10 showed a moderate negative correlation (*χ* ^2^ = 48.881, *p* < 0.001, *r* = −0.348), suggesting that products lacking this information are more likely to be non‐compliant. Criterion C14 also revealed a significant negative correlation (*χ* ^2^ = 53.146, *p* < 0.001, *r* = −0.363), implying that warnings intended for at‐risk groups are often omitted in non‐compliant products. Additionally, C15 (*χ* ^2^ = 25.688, *p* < 0.001, *r* = −0.252) and C16 (*χ* ^2^ = 26.395, *p* < 0.001, *r* = −0.256) both showed significant negative associations, highlighting that the absence of ingredient sourcing and registration information is more prevalent among non‐compliant supplements. Collectively, these findings emphasize the predictive value of specific labeling components in assessing regulatory compliance in dietary supplement products.

**TABLE 8:** Association between regulatory labeling criteria and the compliance of creatine‐based dietary supplements.

Criterion (C)	*χ* ^2^	*p*	Spearman's coefficient (*r*)
Category “Dietary supplement based on…” is mentioned (C4)	47.753	< 0.001	−0.344
Energy value is specified (C10)	48.881	< 0.001	−0.348
Warning “Keep out of reach of children” is present (C14)	53.146	< 0.001	−0.363
Warnings or indications for at‐risk groups (e.g., pregnant women, children) are mentioned (C15)	25.688	< 0.001	−0.252
Registration number (DMP‐MHPS) is stated (C16)	26.395	< 0.001	−0.256

## 4 Discussion

Labeling is a key component in ensuring the safe use of dietary supplements. It provides mandatory and voluntary information on product composition, usage, health and nutrition claims, and safety precautions. Under Regulation (EU) No. 1169/2011, labels must include ingredients, nutrient content, storage and usage instructions, warnings, and manufacturer identity (EPCEU 2011). Claims must not mislead or suggest disease prevention or treatment (Baelde 2008; Brody 2016). However, many supplements make unproven claims, with limited or no demonstrated efficacy outside of specific uses (Cynober 2022; Crenn 2020). Some can cause adverse effects, especially in vulnerable groups like children, pregnant women, and the elderly (Crenn 2020). Studies have also revealed inconsistencies between labeled and actual contents, raising concerns about safety and traceability (Bensa et al. 2023). Among 403 dietary supplements sold outside the pharmaceutical sector in the Beni Mellal‐Khenifra region, labeling non‐compliance was widespread, with an overall rate of 81.4%. This significant figure highlights major shortcomings in labeling accuracy, information transparency, and adherence to regulatory standards established by competent authorities (Food and Drug Administration [FDA] 2005; European Food Safety Authority [EFSA] 2017; European Parliament and Council of the European Union [EPCEU] 2006, 2011; MHSP & MASFRDWF 2023). These findings align with global trends that consistently show dietary supplements, particularly those marketed for sports use, often evade rigorous regulatory oversight, especially when imported or distributed outside official pharmaceutical channels (French Agency for Food, Environmental and Occupational Health, and Safety 2016). In our study, a strong correlation was observed between non‐compliance and the foreign origin of the products, likely due to insufficient border controls and the proliferation of counterfeit supplements in the Moroccan market, as well as the lack of enforcement of traceability regulations for imported products (Es‐Safi et al. 2021). Similarly, Banović Fuentes et al. (2025) analyzed regulatory alerts from the FDA and Health Canada issued between 2005 and 2013 and found that imported supplements, particularly those sold online for sexual enhancement, weight loss, or bodybuilding, were more frequently linked to contamination risks and regulatory violations. Although the high rate of non‐compliance observed in this study highlights significant regulatory weaknesses, this finding should be interpreted with nuance. Some countries with stringent regulatory frameworks demonstrate substantially higher compliance rates, indicating that the risk associated with imported dietary supplements is not solely attributable to their foreign origin, but also to the regulatory standards enforced in the country of export (Bensa et al. 2023). In the Moroccan context, the registration of dietary supplements with the DMP emerges as a critical regulatory measure. This process not only formalizes market authorization but also ensures product traceability, transparency of composition and origin, validation of health claims, and clear identification of manufacturers. Such regulatory oversight is indispensable for reinforcing nutrivigilance and facilitating the timely withdrawal of products that present confirmed health risks, especially among imported items (MHSP & MASFRDWF 2023). The high non‐compliance rate observed in our sample must therefore be viewed within an international context of growing concern regarding the safety of imported supplements, particularly those from regions with weaker oversight. Strengthening national regulatory mechanisms represents a strategic lever for improving the quality, safety, and accountability of dietary supplements on the Moroccan market. In line with this objective, ANSES recently recommended revising the decree of September 26, 2016, to better regulate the list of substances with nutritional or physiological purposes and their conditions of use (ANSES 2024). This recommendation highlights the critical importance of robust and transparent labeling that is clear, understandable, and easily accessible to consumers, thereby minimizing the risk of misuse and enhancing public health protection through rigorous regulatory compliance. The analysis of 94 creatine‐based dietary supplement products in the study area revealed that none complied with regulatory standards related to labeling and traceability. Considering the widespread consumption of creatine among athletes, this finding raises concerns about the potential exposure of a large user population to adverse health effects, thus constituting a serious public health issue (Escalante et al. 2022). Notably, the ANSES has issued warnings regarding the risks associated with such supplements, including hepatic, renal, cardiovascular, and neuropsychological side effects, as well as the possible presence of undeclared banned substances (French Agency for Food, Environmental and Occupational Health, and Safety 2016; Walpurgis et al. 2020). Our findings also reflect a broader trend of unregulated product circulation, often occurring without medical oversight. This issue is further supported by previous studies that have highlighted problems such as inaccurate labeling, variable product composition, and the absence of third‐party certification (Baume et al. 2006; Molina Juan et al. 2021). The analysis of labeling criteria associated with dietary supplement compliance revealed that the most frequent non‐conformities concern the omission of essential product information, particularly the failure to disclose the composition, including the active substance or main ingredient. This element is crucial for informing consumers and ensuring transparency (MHSP & MASFRDWF 2023). The absence of such information leads to terminological ambiguity, undermining informed decision‐making and responsible consumption (Moreira et al. 2019). Additional shortcomings were noted, including the absence of a registration number with the Moroccan DMP, a key element for ensuring product traceability, health surveillance, and risk management (MHSP & MASFRDWF 2023). Other common deficiencies included the absence of mandatory safety warnings and incomplete nutritional information. These observations are consistent with several studies, which highlight their detrimental effects on consumer safety and the overall effectiveness of health protection systems (Mason et al. 2007; Kanter et al. 2018; Domínguez et al. 2021; Agarwal et al. 2022). A comparable situation has been observed in Poland, where suppliers are legally required to notify dietary supplements to the Chief Sanitary Inspector and submit a sample label before commercialization (Kowalska et al. 2019). Despite this regulatory obligation, the rapid growth of the Polish market has exceeded the capacities of official monitoring agencies, resulting in a high incidence of non‐compliance—particularly through adulteration and misleading claims. The convergence of data from both the Moroccan and Polish contexts highlights the urgent need for reinforced health inspection systems and systematic product registration as essential foundations for safeguarding public health and strengthening regulatory oversight. These findings call for urgent strengthening of regulatory controls, better harmonization of labeling standards, restriction of uncertified channels, and greater awareness among consumers and professionals (Farooq and Master 2025). While creatine monohydrate remains safe and effective within a regulated framework (EFSA 2017), the proliferation of non‐compliant and misleading products on the market constitutes both food fraud and a major public health threat (Rodríguez‐Hernández et al. 2024; Zovi et al. 2024).

## 5 Conclusion

The present study reveals a worrying level of non‐compliance among dietary supplements, particularly proteins, amino acids, vitamins, and plant extracts, especially those from informal or unregulated sources. Labeling irregularities, lack of traceability, and discrepancies in declared composition pose significant health risks, particularly for athletes who use such products frequently. Creatine‐based supplements show the highest non‐compliance rates, reinforcing our findings in 2024 (Galman et al. 2024). These results highlight the urgent need for improved regulation, particularly regarding imported and uncertified products. To address these issues, the study recommends: strengthening national registration and certification of sports supplements, enhancing border controls to prevent entry of non‐compliant imports, establishing a WHO and ANSES‐aligned nutrivigilance system for adverse effect monitoring, launching public awareness campaigns targeting consumers and professionals, conducting analytical testing of creatine‐based products to detect banned or harmful substances, initiating clinical follow‐up of creatine users to track health impacts, and providing ongoing training for health professionals, coaches, and retailers. This integrated approach, combining regulation, surveillance, education, and research, is essential to protect consumers and restore confidence in Morocco's dietary supplement market.

## Author Contributions

Conceptualization, A.G. and K.B.; methodology, A.G., M.C., H.A., M.R.K., R.L. and H.B.; software, A.G., M.K., C.D. and H.A.; validation, M.B., F.A.N., K.B. and A.A.Q.; formal analysis, M.B., M.C. and K.B.; investigation, A.G. and K.B.; resources, M.A., N.M. and H.B.; data curation, M.K., C.D. and A.G.; writing – original draft preparation, A.G. and H.A.; writing – review and editing, M.B., K.B., F.A.N., M.R.K., M.C., R.L., H.A.R., and A.A.Q.; visualization, M.A. and K.B.; supervision, K.B.; funding acquisition, F.A.N. and A.A.Q. All authors have read and agreed to the published version of the manuscript.

## Ethics Statement

The authors have nothing to report.

## Consent

Approval for this study was obtained from the relevant authorities at various administrative levels prior to its initiation. In addition, consent was obtained from participants (owners of dietary supplement retail outlets) after explaining that the observation of product labeling would remain anonymous and confidential. It was also clarified that the purpose of the study was to enhance the understanding and safety of dietary supplement consumption in Morocco. Participants were encouraged to provide honest and objective responses to ensure the reliability of the findings.

## Conflicts of Interest

The authors declare no conflicts of interest.

## Data Availability

The data that support the findings of this study are available from the corresponding author upon reasonable request.

## References

[fsn371512-bib-0001] Abreu, R. , C. B. Oliveira , J. A. Costa , J. Brito , and V. H. Teixeira . 2023. “Effects of Dietary Supplements on Athletic Performance in Elite Soccer Players: A Systematic Review.” Journal of the International Society of Sports Nutrition 20, no. 1: 2236060. 10.1080/15502783.2023.2236060. 37462346 PMC10355692

[fsn371512-bib-0002] Agarwal, D. , P. Ravi , B. Purohit , and H. Priya . 2022. “The Effect of Energy and Fat Content Labeling on Food Consumption Pattern: A Systematic Review and Meta‐Analysis.” Nutrition Reviews 80, no. 3: 453–466. 10.1093/nutrit/nuab035. 34339509

[fsn371512-bib-0003] Allali, F. 2017. “Nutrition Transition in Morocco.” Integrative Journal of Medical Sciences 4: 7120–7173. 10.15342/ijms.v4is.145.

[fsn371512-bib-0004] ANSES . 2024. “Opinion on the Update of the Decree of 26 September 2016 Establishing the List of Substances With Nutritional or Physiological Purposes Authorized in Food Supplements and the Conditions of Their Use.” Agence Nationale de Sécurité Sanitaire. https://www.anses.fr/sites/default/files/NUT2023‐SA‐0216.pdf.

[fsn371512-bib-0005] Baelde, D. 2008. “Actualités de la Législation Européenne en Matière D'aliments et D'allégations Santé.” Annales Pharmaceutiques Françaises 66, no. 5–6: 296–299. 10.1016/j.pharma.2008.06.004. 19061729

[fsn371512-bib-0006] Baharirad, N. , A. Naghipour , A. Soroush , et al. 2025. “Prevalence and Pattern of Dietary Supplement Use in Bodybuilding Athletes.” Current Nutrition & Food Science 21, no. 1: 77–83. 10.2174/0115734013282951240226061941.

[fsn371512-bib-0007] Banović Fuentes, J. , I. Beara , and L. Torović . 2025. “Regulatory Compliance of Health Claims on Omega‐3 Fatty Acid Food Supplements.” Food 14, no. 1: 67. 10.3390/foods14010067. PMC11719789 39796357

[fsn371512-bib-0008] Bardou‐Boisnier, S. , and K. Caillaud . 2015. “Les Dispositifs Informationnels Sur Les Compléments Alimentaires: Une Affaire de Santé Publique [International Devices on Food Supplements: A Public Issue].” Questions de Communication 27: 79–104. 10.4000/questionsdecommunication.9705.

[fsn371512-bib-0009] Baume, N. , N. Mahler , M. Kamber , P. Mangin , and M. Saugy . 2006. “Research of Stimulants and Anabolic Steroids in Dietary Supplements.” Scandinavian Journal of Medicine & Science in Sports 16: 41–48. 10.1111/j.1600-0838.2005.00442.x. 16430680

[fsn371512-bib-0010] Bensa, M. , I. Vovk , and V. Glavnik . 2023. “Resveratrol Food Supplement Products and the Challenges of Accurate Label Information to Ensure Food Safety for Consumers.” Nutrients 15, no. 2: 474. 10.3390/nu15020474. 36678345 PMC9861762

[fsn371512-bib-0011] Brody, T. 2016. “Food and Dietary Supplement Package Labeling Guidance From FDA'S Warning Letters and Title 21 of the Code of Federal Regulations.” Comprehensive Reviews in Food Science and Food Safety 15, no. 1: 92–129. 10.1111/1541-4337.12172. 33371576

[fsn371512-bib-0012] Chiba, T. , Y. Sato , S. Suzuki , and K. Umegaki . 2015. “Concomitant Use of Dietary Supplements and Medicines in Patients due to Miscommunication With Physicians in Japan.” Nutrients 7, no. 4: 2947–2960. 10.3390/nu7042947. 25894658 PMC4425182

[fsn371512-bib-0013] Crenn, P. 2020. “Bénéfices et Risques des Compléments Alimentaires.” Nutrition Clinique Et Métabolisme 34, no. 3: 201–206. 10.1016/j.nupar.2020.04.006.

[fsn371512-bib-0014] Cynober, L. 2022. “Bienfaits et Méfaits des Compléments Alimentaires.” Bulletin de l'Académie Nationale de Médecine 206, no. 5: 660–666. 10.1016/j.banm.2022.02.014.

[fsn371512-bib-0015] Devaux, S. , and M. Brisard . 2016. “Consommation de Compléments Alimentaires Chez les Triathlètes: Résultats D'une Enquête Régionale.” Nutrition Clinique et Métabolisme 30, no. 2: 118. 10.1016/j.nupar.2016.04.031.

[fsn371512-bib-0016] Domínguez, L. , V. Fernández‐Ruiz , P. Morales , M.‐C. Sánchez‐Mata , and M. Cámara . 2021. “Assessment of Health Claims Related to Folic Acid in Food Supplements for Pregnant Women According to the European Regulation.” Nutrients 13, no. 3: 937. 10.3390/nu13030937. 33799440 PMC7998675

[fsn371512-bib-0017] EFSA . 2017. “Dietary Reference Values for Nutrients: Summary Report (EFSA Supporting Publication).” European Food Safety Authority 14, no. 12: e15121. 10.2903/sp.efsa.2017.e15121.

[fsn371512-bib-0018] El Kartouti, A. , and Y. Khalfaoui . 2020. “The Profile of Consumers of Food Supplements in Morocco.” Saudi Journal of Medicine & Medical Sciences 8, no. 3: 106–112. 10.36348/sjtcm.2020.v03i06.002.

[fsn371512-bib-0019] EPCEU . 2002. “Directive 2002/46/EC of the European Parliament and of the Council on the Approximation of the Laws of the Member States Relating to Food Supplements.” Official Journal of the European Union L183: 51–57. https://eur‐lex.europa.eu/legal‐content/EN/TXT/?uri=CELEX%3A32002L0046.

[fsn371512-bib-0020] EPCEU . 2006. “Regulation (EC) no 1924/2006 of the European Parliament and of the Council of 20 December 2006 on Nutrition and Health Claims Made on Foods.” EUR‐Lex. https://eur‐lex.europa.eu/legal‐content/EN/TXT/?uri=CELEX%3A32006R1924.

[fsn371512-bib-0021] EPCEU . 2011. “Regulation (EU) No. 1169/2011 of the European Parliament and of the Council of 25 October 2011 on the Provision of Food Information to Consumers.” EUR‐Lex. https://eur‐lex.europa.eu/legal‐content/EN/TXT/?uri=CELEX%3A32011R1169.

[fsn371512-bib-0022] Escalante, G. , A. M. Gonzalez , D. St Mart , et al. 2022. “Analysis of the Efficacy, Safety, and Cost of Alternative Forms of Creatine Available for Purchase on Amazon.com: Are Label Claims Supported by Science?” Heliyon 8, no. 12: e12113. 10.1016/j.heliyon.2022.e12113. 36544833 PMC9761713

[fsn371512-bib-0023] Es‐Safi, I. , H. Mechchate , A. Amaghnouje , et al. 2021. “Marketing and Legal Status of Phytomedicines and Food Supplements in Morocco.” Journal of Complementary and Integrative Medicine 18, no. 2: 279–285. 10.1515/jcim-2020-0168. 32950964

[fsn371512-bib-0024] Farooq, S. , and V. A. Master . 2025. “Food and Dietary Supplements.” In Handbook for Designing and Conducting Clinical and Translational Research: Translational Urology, edited by A. E. M. Eltorai , A. Arab , A. Atala , and M. M. Siddiqui , 365–368. Academic Press. 10.1016/B978-0-323-90186-4.00019-5.

[fsn371512-bib-0025] Food and Agriculture Organization & World Health Organization . 2024. “General Standard for the Labelling of Pre‐Packaged Foods: CXS 1–1985 (Revised in 2018 and 2024).” FAO & WHO. https://www.fao.org/fao‐who‐codexalimentarius/codex‐texts/list‐standards.

[fsn371512-bib-0026] Food and Drug Administration . 2005. “Dietary Supplement Labeling Guide.” U.S. Department of Health and Human Services, FDA. https://www.fda.gov/food/dietary‐supplements/dietary‐supplement‐labeling‐guide.

[fsn371512-bib-0027] French Agency for Food, Environmental and Occupational Health & Safety . 2016. “Opinion on the Risks Associated With the Consumption of Food Supplements for Athletes Seeking to Develop Muscle or Reduce Body Fat.” ANSES. https://www.anses.fr/en/system/files/NUT2014SA0008EN.pdf.

[fsn371512-bib-0028] Galman, A. , M. Chikhaoui , M. Bouhrim , et al. 2024. “Fitness and Dietary Supplements: A Cross‐Sectional Study on Food Practices and Nutrivigilance.” Nutrients 16, no. 22: 3928. 10.3390/nu16223928. 39599714 PMC11597613

[fsn371512-bib-0029] Grand View Research . 2023. “Sports Nutrition Market Size, Share & Trends Report, 2024–2030.” GVR. https://www.grandviewresearch.com/industry‐analysis/sports‐nutrition‐market.

[fsn371512-bib-0030] Hasan, M. , S. S. Allehdan , T. Alalwan , S. Perna , and R. Tayyem . 2024. “Overview of Dietary Supplements Use: A Narrative Review.” Current Nutrition & Food Science 20, no. 8: 973–981. 10.2174/0115734013271923231227041108.

[fsn371512-bib-0031] Hassan, S. , C. Egbuna , H. Tijjani , et al. 2020. “Dietary Supplements: Types, Health Benefits, Industry and Regulation.” In Functional Foods and Nutraceuticals, edited by C. Egbuna and G. Dable Tupas , 23–38. Springer. 10.1007/978-3-030-42319-3_3.

[fsn371512-bib-0032] Kanter, R. , L. Vanderlee , and S. Vandevijvere . 2018. “Front‐of‐Package Nutrition Labelling Policy: Global Progress and Future Directions.” Public Health Nutrition 21, no. 8: 1399–1408. 10.1017/S1368980018000010. 29559017 PMC10261279

[fsn371512-bib-0033] Kowalska, A. , M. Bieniek , and L. Manning . 2019. “Food Supplements' Non‐Conformity in Europe—Poland: A Case Study.” Trends in Food Science & Technology 93: 262–270. 10.1016/j.tifs.2019.09.022.

[fsn371512-bib-0034] Kozhuharov, V. R. , K. Ivanov , and S. Ivanova . 2022. “Dietary Supplements as Source of Unintentional Doping.” BioMed Research International 2022, no. 1: 8387271. 10.1155/2022/8387271. 35496041 PMC9054437

[fsn371512-bib-0035] Mah, E. , O. Chen , D. J. Liska , and J. B. Blumberg . 2022. “Dietary Supplements for Weight Management: A Narrative Review of Safety and Metabolic Health Benefits.” Nutrients 14, no. 9: 1787. 10.3390/nu14091787. 35565754 PMC9099655

[fsn371512-bib-0036] Marcotrigiano, V. , C. Lanzilotti , D. Rondinone , et al. 2018. “Food Labelling: Regulations and Public Health Implications.” Annali dell'Istituto Superiore di Sanità 30, no. 3: 220–228. 10.7416/ai.2018.2213. 29670991

[fsn371512-bib-0037] Mason, M. J. , D. L. Scammon , and X. Fang . 2007. “The Impact of Warnings, Disclaimers, and Product Experience on Consumers' Perceptions of Dietary Supplements.” Journal of Consumer Affairs 41, no. 1: 74–99. 10.1111/j.1745-6606.2006.00069.x.

[fsn371512-bib-0038] Maughan, R. J. , L. M. Burke , J. Dvorak , et al. 2018. “IOC Consensus Statement: Dietary Supplements and the High‐Performance Athlete.” International Journal of Sport Nutrition and Exercise Metabolism 28, no. 2: 104–125. 10.1123/ijsnem.2018-0020. 29589768

[fsn371512-bib-0039] Ministry of Agriculture, Sea Fishing, Rural Development, Water and Forests . 2009. “Law No. 25‐08 Establishing the National Office for Food Safety (ONSSA). Official Bulletin of Morocco, No. 5714.” MASFRDWF. https://www.onssa.gov.ma/wp‐content/uploads/2022/06/Reglementation/A.Reglementation‐Transversale/1.%20Missions%20et%20Attributions%20de%20ONSSA/LOI.25‐08.FR.pdf.

[fsn371512-bib-0040] MHSP & MASFRDWF . 2023. “Circulaire N° 834 du 14/11/2023 Relative Aux Denrées Alimentaires et Boissons Destinées à Une Alimentation Particulière.” Ministry of Health and Social Protection & Ministry of Agriculture, Sea Fishing, Rural Development, Water and Forests. https://dmp.sante.gov.ma/actualites/details/circulaire‐n‐834‐du‐14‐11‐2023‐relative‐auxdenrees‐alimentaires‐et‐boissons‐destinees‐a‐une‐alimentation‐particuliere.

[fsn371512-bib-0041] MHSP . 1974. “Decree No. 2‐72‐373 Establishing a National Laboratory for the Control of Medicines and Pharmaceutical Specialties (Directorate of Medicines and Pharmacy).” Ministry of Health and Social Protection, Morocco. https://www.sante.gov.ma/Pages/ADM_Centrale/DMP.aspx.

[fsn371512-bib-0042] Molina Juan, L. , I. Sospedra , A. Perales , C. González‐Díaz , A. Gil‐Izquierdo , and J. M. Martínez‐Sanz . 2021. “Analysis of Health Claims Regarding Creatine Monohydrate Present in Commercial Communications for a Sample of European Sports Foods Supplements.” Public Health Nutrition 24, no. 4: 632–640. 10.1017/S1368980020005121. PMC11574825 33468268

[fsn371512-bib-0043] Moreira, M. J. , J. García‐Díez , J. M. M. M. De Almeida , and C. Saraiva . 2019. “Evaluation of Food Labelling Usefulness for Consumers.” International Journal of Consumer Studies 43, no. 4: 327–334. 10.1111/ijcs.12511.

[fsn371512-bib-0044] Rodríguez‐Hernández, M. D. , Á. Gil‐Izquierdo , C. J. García , et al. 2024. “Health Claims for Sports Drinks Analytical Assessment According to European Food Safety Authority's Scientific Opinion.” Nutrients 16, no. 13: 1980. 10.3390/nu16131980. 38999728 PMC11243318

[fsn371512-bib-0045] Tarn, D. M. , D. A. Paterniti , J. S. Good , et al. 2013. “Physician–Patient Communication About Dietary Supplements.” Patient Education and Counseling 91, no. 3: 287–294. 10.1016/j.pec.2013.01.021. 23466249 PMC3648214

[fsn371512-bib-0046] Touijri, O. , B. Al Ibrahmi , Y. Nahab , A. Quyou , and M. L. Ouaidi . 2024. “Profile of Dietary Supplement Consumers in the Region of Rabat–Salé–Kénitra, Morocco.” International Journal of Tropical Medicine 18, no. 4: 1857. 10.4081/itjm.2024.1857.

[fsn371512-bib-0047] Walpurgis, K. , A. Thomas , H. Geyer , U. Mareck , and M. Thevis . 2020. “Dietary Supplement and Food Contaminations and Their Implications for Doping Controls.” Food 9, no. 8: 1012. 10.3390/foods9081012. PMC7466328 32727139

[fsn371512-bib-0048] Walrand, S. 2018. “Dietary Supplement Intake Among the Elderly: Hazards and Benefits.” Current Opinion in Clinical Nutrition and Metabolic Care 21, no. 6: 465–470. 10.1097/MCO.0000000000000512. 30239340

[fsn371512-bib-0049] Wang, X. , H. Wang , and H. Wu . 2023. “Dietary and Ergogenic Supplementation to Improve Elite Swimming Players' Performance and Recovery.” Science & Sports 38, no. 5–6: 510–518. 10.1016/j.scispo.2022.12.004.

[fsn371512-bib-0050] Zovi, A. , A. Vitiello , M. Sabbatucci , et al. 2024. “Food Supplements Marketed Worldwide: A Comparative Analysis Between the European and the U.S. Regulatory Frameworks.” Journal of Dietary Supplements 22, no. 1: 25–40. 10.1080/19390211.2024.2389397. 39221704

